# Physiological and Defense Responses of Tea Plants to Elevated CO_2_: A Review

**DOI:** 10.3389/fpls.2020.00305

**Published:** 2020-03-20

**Authors:** Golam Jalal Ahammed, Xin Li, Airong Liu, Shuangchen Chen

**Affiliations:** ^1^College of Forestry, Henan University of Science and Technology, Luoyang, China; ^2^Key Laboratory of Tea Quality and Safety Control, Ministry of Agriculture, Tea Research Institute, Chinese Academy of Agricultural Sciences, Hangzhou, China

**Keywords:** climate change, tea quality, elevated CO_2_, secondary metabolism, catechin, theanine, caffeine, plant defense

## Abstract

Rising atmospheric carbon dioxide, an important driver of climate change, has multifarious effects on crop yields and quality. Despite tremendous progress in understanding the mechanisms of plant responses to elevated CO_2_, only a few studies have examined the CO_2_-enrichment effects on tea plants. Tea [*Camellia sinensis* (L.)], a non-deciduous woody perennial plant, operates massive physiologic, metabolic and transcriptional reprogramming to adapt to increasing CO_2_. Tea leaves elevate photosynthesis when grown at CO_2_–enriched environment which is attributed to increased maximum carboxylation rate of RuBisCO and maximum rates of RuBP regeneration. Elevated CO_2_-induced photosynthesis enhances the energy demand which triggers respiration. Stimulation of photosynthesis and respiration by elevated CO_2_ promotes biomass production. Moreover, elevated CO_2_ increases total carbon content, but it decreases total nitrogen content, leading to an increased ratio of carbon to nitrogen in tea leaves. Elevated CO_2_ alters the tea quality by differentially influencing the concentrations and biosynthetic gene expression of tea polyphenols, free amino acids, catechins, theanine, and caffeine. Signaling molecules salicylic acid and nitric oxide function in a hierarchy to mediate the elevated CO_2_-induced flavonoid biosynthesis in tea leaves. Despite enhanced synthesis of defense compounds, tea plant defense to some insects and pathogens is compromised under elevated CO_2_. Here we review the physiological and metabolic responses of tea plants to elevated CO_2_. In addition, the potential impacts of elevated CO_2_ on tea yield and defense responses are discussed. We also show research gaps and critical research areas relating to elevated CO_2_ and tea quality for future study.

## Introduction

Increasing atmospheric CO_2_ is the most prominent driver of global warming. At present, global atmospheric CO_2_ concentration is 407.65 ppm (recorded in September 2019^1^), which was only 270 ppm during the preindustrial era. Over the last 200 years, such an unparalleled increase in atmospheric CO_2_ occurred due to massive human anthropogenic activities such as deforestation, fossil-based fuel combustion, rapid urbanization and industrialization (Ahuja et al., 2010). The concentration of atmospheric CO_2_ is still increasing, and it will possibly reach 800 ppm by the end of the 21st century (IPCC, 2014). It is inferred that with increasing CO_2_, the adversities of extreme climate events such as heatwave, drought, and frost will be increasing, which will variously affect tea yield, quality and ecosystem (Li et al., 2018, 2019a; Ahmed et al., 2019). Nonetheless, tea plantations play a significant role in CO_2_ sequestration and thus tea gardens can be useful in mitigating global warming (Pramanik and Phukan, 2020).

Tea is the most popular beverage consumed across the seven continents (Macfarlane and Macfarlane, 2004), even though its cultivation is limited to Asia and Africa. Green tea is basically manufactured from the species *Camellia sinensis* (L.) Kuntze through rapid roasting of fresh leaves to avoid oxidation (Han et al., 2016). The popularity of green tea is increasing day by day, not only for its pleasant flavor but also for numerous health benefits such as anti-inflammatory, anti-cancer, anti-obesity, and anti-allergic effects on humans (Kim et al., 2009; Siamwala et al., 2013; Mancini et al., 2017). Due to the increasing demand for tea, areas belonging to tea cultivation are increasing in tea growing regions including China (Han et al., 2016, 2018). Tea is a long-living commercial beverage crop that can remain productive for a century if the gardens are well managed. The long life span of tea plants compels them to face environmental challenges years after years through physiological adaptations to changing climate (Larson, 2015; Li et al., 2017). Drivers of climate change differentially affect tea yield and quality on a spatiotemporal basis (Wijeratne et al., 2007; Han et al., 2016). Although the effects of climate change on the yield of food crops have extensively been studied, its impact on tea has received less attention. In particular, research on the effect of elevated CO_2_ on tea is still in its infancy (Ahmed et al., 2019). However, recently more attention has been paid to the issue and inter-governmental initiatives have been taken to address climate change effects on tea under the umbrella of the Food and Agriculture Organization of the United Nations (Han et al., 2018). Several research papers on the effect of CO_2_ on tea yield and quality were published in last 3 years (Hui et al., 2016; Li et al., 2016, 2017, 2018, 2019a; Roy et al., 2019; Pramanik and Phukan, 2020). However, a comprehensive review of the effect of elevated CO_2_ on tea plants is still missing. In this review, we intend to summarize key physiological and metabolic processes associated with the tea quality in response to elevated CO_2_. Besides, the potential impact of elevated CO_2_ on tea yield and defense has been discussed. We also try to find out research gaps and critical research areas on the effect of elevated CO_2_ on tea quality for future study.

## Growth and Basic Physiological Responses to Elevated Co_2_ in Tea Plants

Evidence from a number of studies shows that elevated CO_2_ improves leaf number, leaf area index, branches, shoot length, root length, and overall biomass accumulation in C_3_ plants (Huang et al., 2007; Kimball, 2016). Similarly, exposure of tea plants to elevated CO_2_ (800 μmol mol^–1^) even for 24 days increases plant height, shoot dry weight and root dry weight (Li et al., 2017). Recently, Li et al. (2019a) also showed that exposure of the 1-year-old tea seedlings to 770.5 μmol mol^–1^ CO_2_ concentration in open-top chambers for 60 days significantly increases biomass accumulation in terms of fresh weights of leaves (+15.04%), roots (+22%), and whole plants (+16.26%). Since the yield of tea is the sum of buds and young leaves, elevated CO_2_-induced promotion in shoot biomass greatly contributes to tea yield.

**FIGURE 1:**
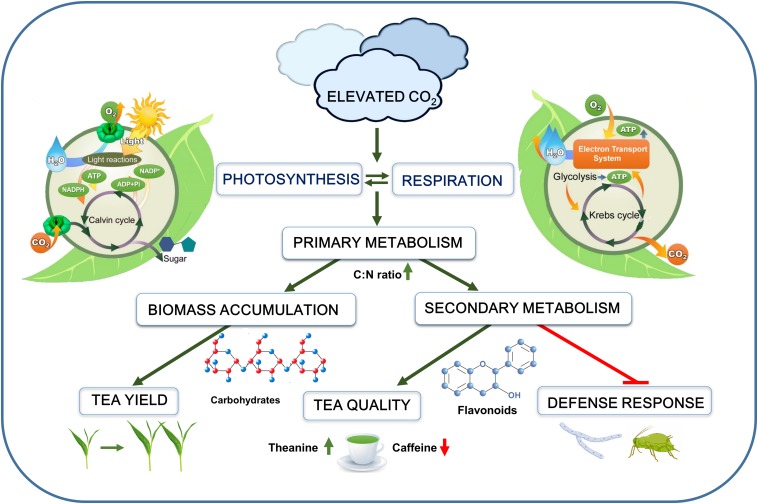
Schematic representation of the overall effect of elevated CO_2_ on tea plants. Elevated CO_2_ increases photosynthesis and respiration, leading to enhanced primary metabolism. Stimulation of primary metabolism increases biomass accumulation as well as carbon-flow toward secondary metabolic pathways. Elevated CO_2_ increases concentrations of carbohydrates (starch and sugars), flavonoids, theanine, and salicylic acid, but it decreases caffeine content in tea leaves. Despite enhanced production of secondary metabolites and defense compounds, tea plant defense against aphids and necrotrophic fungi is greatly compromised under elevated CO_2_. The green arrows indicate promotion, while the red arrows or blunt arrow-heads indicate inhibition.

In C_3_ plants, such as tea, elevated CO_2_ stimulates the CO_2_ assimilation rate by providing sufficient substrates (i.e., CO_2_) required for photosynthetic reactions (Figure 1). This eventually leads to the enhanced supply of energy-rich compounds, such as adenosine triphosphate (ATP) and nicotinamide adenine dinucleotide phosphate (NADPH) under elevated CO_2_. Notably, stomata as a basic channel for gas exchange plays an important role in CO_2_ and O_2_ exchange between plant and atmosphere. A leaf gas exchange analysis showed that exposure of tea seedlings to elevated CO_2_ for 60 days increases the net photosynthetic rate (+20%) and intercellular CO_2_ concentrations (+15.74%); however, it decreases the stomatal conductance (−5.52%) and transpiration rate (−9.40%) in tea leaves (Li et al., 2019a). Intriguingly, the increase in net photosynthetic rate is much higher (+87.9%) in case of short duration (24 days) CO_2_-enrichment treatment, suggesting a potential photosynthetic acclimation following prolonged exposure to elevated CO_2_ in tea plants (Li et al., 2017). This raises the question of how tea plants increase the photosynthetic rate under reduced stomatal conductance. In C_3_ plants, the activity of ribulose-1,5-bis-phosphate (RuBP) carboxylase/oxygenase (RuBisCO) is critical for CO_2_ assimilation (Eisenhut et al., 2019; Thomey et al., 2019). Elevated CO_2_ increases maximum carboxylation rate of RuBisCO and maximum rates of RuBP regeneration in tea plants (Li et al., 2017), thus facilitating carboxylation over oxygenation of RuBP (Amthor, 1997; Li et al., 2013), which potentially contributes to increased CO_2_ assimilation in tea leaves (Li et al., 2017). Although exposure of tea plants to elevated CO_2_ (648–65 μmol mol^–1^) for 45 days does not significantly affect photosynthetic pigment content (Hui et al., 2016), a 3-month long CO_2_ enrichment (750 μmol mol^–1^) results in 18.4, 22.0, and 20.1% increased chlorophyll *a*, chlorophyll *b*, and carotenoid concentrations in tea shoots (Jiang et al., 2005). Through respiration, a part of the carbon is consumed by leaves, buds, shoots, and roots, and the rest is released as CO_2_ via stomata to the atmosphere (Huang et al., 2007; Eisenhut et al., 2019). Ultimately, respiration supplies energy in the form of ATP to plant cells. In tea plants, elevated CO_2_ (800 μmol mol^–1^) promotes total respiration rate (+28.9–53.6%), which is attributed to concurrent increases in the salicylhydroxamic acid (SHAM)-resistant respiration as well as the cyanide (CN)-resistant respiration (Li et al., 2017), suggesting that elevated CO_2_ triggers both electron transport pathways, i.e., the ATP-coupling SHAM-resistant cytochrome pathway and the CN-resistant alternative pathway utilized by plant mitochondria. Notably, the alternative respiration greatly contributes to redox homeostasis by minimizing the excess production of reactive oxygen species via the mitochondrial electron transport chain (Gong et al., 2020). It is believed that elevated CO_2_-induced photosynthesis enhances energy demand which triggers respiration as well.

## Mechanisms of Elevated Co_2_-Induced Changes in Primary and Secondary Metabolism

**FIGURE 2:**
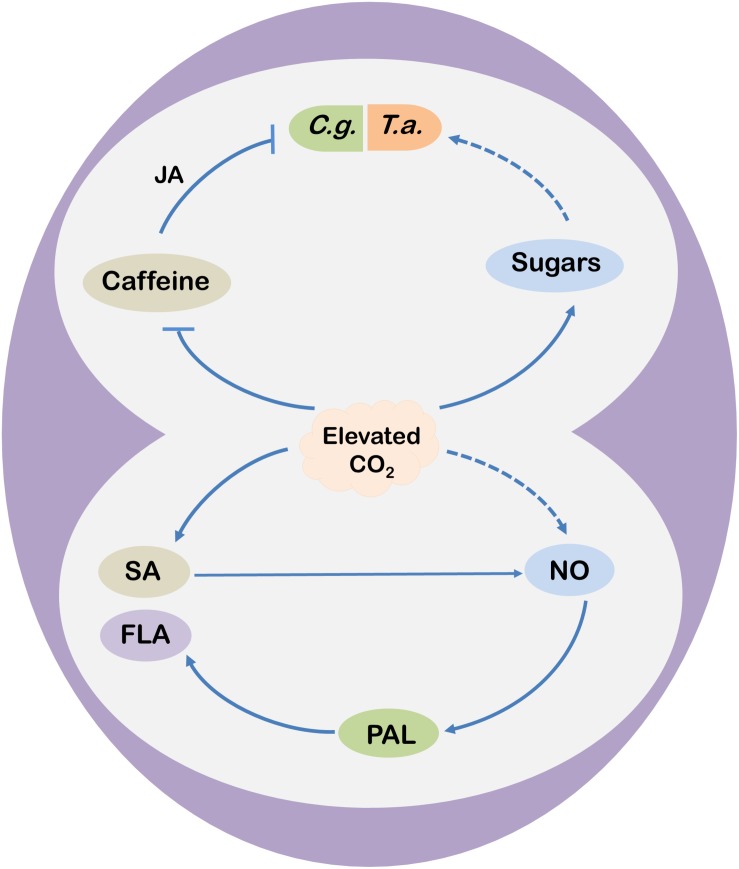
A schematic model showing potential mechanisms of elevated CO_2_-induced changes in tea functional components and defense responses. Elevated CO_2_ enhances salicylic acid (SA) concentrations in tea leaves that trigger nitric oxide (NO) accumulation and subsequent flavonoid (FLA) biosynthesis by stimulating the activity of phenylalanine ammonia-lyase (PAL) under elevated CO_2_ conditions. Nonetheless, elevated CO_2_ may also promote FLA biosynthesis via NO in a SA-independent manner. Meanwhile, elevated CO_2_ inhibits caffeine biosynthesis, leading to reduced accumulation of jasmonic acid (JA) via the lipoxygenase (LOX)-dependent pathway. Decreased JA biosynthesis compromises caffeine-induced resistance to *C. gloeosporioides* (*C. g.*, the anthracnose fungus) in tea plants. On the other hand, elevated CO_2_ increases the concentrations of soluble sugars, soluble proteins, and free fatty acids in tea leaves, which might contribute to the increased population abundance of *T. aurantii* (*T. a.*; the tea aphid) under elevated CO_2_. Arrows indicate promotion, while blunt arrow-heads indicate inhibition. Dotted lines are assumptions.

Among numerous plant metabolites, only a minor portion of metabolites are directly used for plant growth and development, commonly termed as “primary metabolites”; however, numerous other metabolites, commonly termed as “secondary metabolites,” are used for various functions, including plant defense against biotic and abiotic stresses (Zhao et al., 2013, 2019). When tea plants are grown under elevated CO_2_, tea leaves accumulate more soluble sugar, sucrose and starch (Li et al., 2017, 2019a). However, elevated CO_2_ (770.5 μmol mol^–1^) decreases free fatty acid content with no effect on soluble protein content in tea leaves (Li et al., 2019a). In addition to the influence on nutrient compositions, elevated CO_2_ affects functional components of tea leaves. For instance, a 24 days or 60 days exposure of tea plants to elevated CO_2_ significantly increases the concentrations of free amino acids, theanine, and tea polyphenols, but it decreases the content of caffeine in tea leaves (Li et al., 2017, 2019a). Tea polyphenols are the major antioxidant compounds in tea leaves that provide astringency to tea infusion (Chen et al., 2018; Li et al., 2019b). Nonetheless, polyphenols have numerous health benefits, such as relieving inflammation and oxidative stress (Zhao et al., 2016; Du et al., 2018; Fei et al., 2018). Under elevated CO_2_, tea polyphenols such as total catechins (major flavonoids) as well as (−)-epigallocatechin (EGC) and (−)-epigallocatechin-3-gallate (EGCG) concentrations become high (Li et al., 2017). A couple of comprehensive studies on the effect of elevated CO_2_ on tea quality show that elevated CO_2_ upregulates the expression of genes encoding key enzymes required for the biosynthesis of phenylpropanoids that act as precursors of different catechins. More importantly, the transcript levels of *ANTHOCYANIDIN REDUCTASE (CsANR)* remarkably upregulates (>500%) under elevated CO_2_, which encodes specific enzymes that catalyze the conversion of anthocyanidins into epicatechins (Li et al., 2017, 2019a). Interestingly, elevated CO_2_ also increases the concentrations of salicylic acid (SA) in tea leaves compared with the ambient CO_2_ (Li et al., 2017, 2019a). It has been further revealed that SA mediates elevated CO_2_-induced flavonoid biosynthesis in tea leaves and SA acts downstream of CO_2_ and enhances nitric oxide (NO) production to increase flavonoid biosynthesis (Li et al., 2019c). However, a SA-independent NO production pathway may also function under elevated CO_2_ (Figure 2).

Theanine is a kind of non-protein amino acid that comprises about 50% of total amino acid content in tea leaves and it imparts the “umami” taste of tea (Vuong et al., 2011; Cheng et al., 2017). Theanine has numerous health benefits including improvement of memory, reduction of blood pressure, stress relief and stimulation of relaxation (Unno et al., 2017). Studies have revealed that elevated CO_2_ increases theanine concentration in tea leaves (Li et al., 2017, 2019a). However, concentrations of some essential amino acids are differentially modulated under elevated CO_2_ in tea leaves (Li et al., 2017, 2019a). There are also some discrepancies in existing literature relating to the elevated CO_2_ effect on theanine content, which is largely attributed to the exposure duration in different studies. For instance, Li et al. (2017) and Li et al. (2019a) exposed tea seedlings to elevated levels of CO_2_ (800 and 770 μmol mol^–1^) for 24 and 60 days, respectively. They found a significant increase in leaf theanine concentration under elevated CO_2_ conditions. However, Jiang et al. (2006) exposed tea seedlings to elevated CO_2_ (750 μmol mol^–1^) for 6 months and found a significant decrease in leaf theanine content. It is highly likely that long-term exposure to elevated CO_2_ would affect total carbon and nitrogen pools, causing an increase in the C:N ratio, which might also affect the biosynthesis of nitrogenous compounds, including theanine. Likewise, Hui et al. (2016) also found a negative effect of elevated CO_2_ on amino acid (including theanine) content, which eventually increased polyphenols to amino acid ratio in tea leaves.

In addition to flavonoids and theanine, caffeine accumulation also depends on environmental factors (Li et al., 2016, 2017). Studies have revealed that caffeine content declines when tea plants are grown under elevated CO_2_ conditions (Li et al., 2016, 2017, 2019a). Accordingly, transcript analysis of caffeine synthetic pathway genes reveals that elevated CO_2_ remarkably suppresses the expression of *INOSINE 5′-MONOPHOSPHATE DEHYDROGENASE (CsTIDH)*, *s-ADENOSYL-L-METHIONINE SYNTHASE (CssAMS)* and *TEA CAFFEINE SYNTHASE 1 (CsTCS1)* in tea leaves (Li et al., 2017).

## Defense Response of Tea Plants as Influenced by Elevated Co_2_

A general perception is that elevated CO_2_-induced stimulation of primary metabolism promotes secondary metabolism in plants, leading to enhanced synthesis of defense-related compounds (Noctor and Mhamdi, 2017). Although there is no discrepancy regarding the enhancement of SA levels under elevated CO_2_, changes in jasmonic acid (JA) levels were found different in two studies in tea plants. A study that used 2-year-old Longjing 43 tea seedlings showed that exposure of tea plants to elevated CO_2_ (800 μmol mol^–1^) for 14 days significantly decreases JA concentrations (Li et al., 2016); however, recently Li et al. (2019a) reported that exposure of 1-year-old Longjing Changye tea seedlings to elevated CO_2_ (770 μmol mol^–1^) for 60 days significantly increases JA concentrations (+98.6%) in tea leaves (Li et al., 2019a). Despite the increased accumulation of these metabolites under elevated CO_2_, plant resistance to some insects and pathogens is compromised in tea plants when grown under elevated CO_2_ (Li et al., 2019a). This raises another outstanding question, how tea plants balance growth and defense while improving tea quality under high levels of atmospheric CO_2_ concentrations.

Pathogens and insects invade plants in search of nutrients. While pathogens may reside inside plant cells to complete their life-cycle, insects mostly feed on plants. Therefore, palatably of host leaves matters to insect pests, which is largely dependent on the nutrient constituents of the plant organs (Alba et al., 2014). Studies have revealed that elevated CO_2_ alters nutrient constituents as well as defense compounds in leaves, leading to significant changes in insect infestation (Robinson et al., 2012; Li et al., 2019a). Thus the growth and development of herbivorous and sap-sucking insects is indirectly altered by elevated CO_2_ through its direct effects on plant biomass and nutrient compositions (Kazan, 2018; Li et al., 2019a). In general, elevated CO_2_ triggers population growth of sap-sucking insects such as aphids (e.g., *Myzus persicae*), whiteflies (e.g., *Bemisia tabaci*), and planthoppers (e.g., *Nilaparvata lugens*) (Jiang et al., 2016; Li et al., 2019a). However, the growth, survival rates and population density of most leaf-chewing insects are suppressed by elevated CO_2_ possibly because of the potential deterioration of nutrient quality of their feeds (Coll and Hughes, 2008; Kazan, 2018). In a recent study, Li et al. (2019a) showed that the population abundance of tea aphid (*Toxoptera aurantii*) significantly increased (+4.24–41.17%) when these aphids were fed on tea seedlings grown under elevated CO_2_. Although the study concludes that a reduction in caffeine content under elevated CO_2_ is potentially involved in reduced defense against aphids, such a low decrease (−3.38%) in caffeine content questions this claim. Therefore, it is possible that improved leaf nutrient status under elevated CO_2_, i.e., increased soluble sugars, soluble proteins and free amino acids might play a major role in increased tea aphid abundance due to the characteristics features of aphids, such as short life span, high body weight, increased fecundity and population growth under elevated CO_2_. Notably, stomatal closure and subsequent minimization of transpirational water losses can result in improved leaf water status, which promotes the infestation of pea aphid (*Acyrthosiphon pisum*) in *Medicago truncatula* (Sun et al., 2015; Kazan, 2018). As elevated CO_2_ also reduces the transpiration rate in tea plants by regulating stomatal conductance, the leaf water status may play a role in increased tea aphid abundance.

The role of caffeine in tea plant defense against pathogenic fungus has been reported under elevated CO_2_. A reduction in caffeine content in tea leaves under elevated CO_2_ conditions increases the susceptibility of the tea plants to *Colletotrichum gloeosporioides*, which causes anthracnose, brown blight and dieback disease of tea in different geographical locations depending on the weather conditions (Li et al., 2016). Interestingly, exogenous application of caffeine suppresses the necrotic lesions caused by *C. gloeosporioides*, which is attributed to the caffeine-induced elevation of endogenous JA content under elevated CO_2_ conditions in tea leaves. The study also revealed that caffeine-induced increase in JA levels is attributed to JA biosynthesis through the lipoxygenase (LOX) pathway under elevated CO_2_ in tea leaves (Figure 2). Based on this study, it is quite clear that caffeine plays a vital role in tea plant defense against necrotrophic fungal pathogens in tea plants; however, this response is compromised under elevated CO_2_ due to reduced biosynthesis of caffeine and JA (Li et al., 2016). Conversely, elevated CO_2_ improves the resistance of *Arabidopsis thaliana* to the fungal plant pathogen *Plectosphaerella cucumerina* and the oomycete pathogen *Hyaloperonospora arabidopsidis* by stimulating the JA-dependent and SA-dependent defense priming, respectively (Williams et al., 2018).

## Conclusion and Future Perspectives

Surveys of literature show that the effects of elevated CO_2_ on tea were mostly studied by exposing the plants to a range of artificially enriched CO_2_ levels (550, 650, 750, and 800) for various durations (24 days, 45 days, 60 days, and 6 months) in open-top chambers or controlled closed chambers, suggesting that studies using free air CO_2_ enrichment (FACE) for long-duration are needed to better understand the realistic responses of tea plants to climate change. Notably, about a 100-year life span of tea plants allows them to witness and experience gradual changes in atmospheric CO_2_ concentrations in a single generation, which possibly compels the tea plants to operate massive physiologic, metabolic and transcriptional reprogramming to adapt to changing climate. The two levels of CO_2_, 550 and 800 that are frequently used for CO_2_-enrichment studies, are predicted atmospheric CO_2_ concentrations of the year 2050 and 2100, respectively. Thus, it is quite unusual that tea plants will experience such high levels of CO_2_ overnight, which is possibly the main limitation of the existing research. Therefore, it will be more meaningful to explore how small changes in atmospheric CO_2_ levels would affect tea quality.

Climate change is a cumulative effect of multiple factors, such as changes in temperatures, radiation, precipitation, and CO_2_ levels. Thus it is important to study the combined effects of multiple factors along with elevated CO_2_ to better mimic the real-world situations. Availability or deficiency of essential macro and micro elements and their effect on tea quality under elevated CO_2_ conditions can also be considered from the point of nutraceutical value. In this regard, the role of elevated CO_2_ in the regulation of γ-aminobutyric acid (GABA), a non-proteinogenic amino acid in tea with numerous health benefits, should be considered. Moreover, safety factors such as the occurrence of toxicants, mycotoxins, and contaminants in tea under elevated CO_2_ remain largely unknown. In this aspect, biotic factors such as insect herbivory and pathogen infection can also be included. Notably, how the quality of so-called “bug-bitten tea,” a kind of Oolong tea that is produced under special circumstances upon infestation with tea green leafhoppers (*Empoasca vitis*), is influenced by elevated CO_2_, could be an interesting research topic. One factor, which has been ignored in studying elevated CO_2_ effects on tea plants, is the volatile emission. Plants emit multiple volatile compounds upon insect and pathogen attacks. The volatile compounds not only impart characteristic aroma to tea as a quality parameter but also serve as defense signals and media for plant-plant communication as well as plant-insect interactions (Zhao et al., 2019). Therefore, this issue is expected to address in future studies.

When CO_2_ concentration increases in the atmosphere, it not only influences plants but also other animals including insects and pests. Elevated CO_2_ can influence the virulence, aggressiveness, growth, development, fecundity, fitness, and survival of pests and pathogens by altering host physiology (Kazan, 2018). Therefore, it is highly likely that insects and pathogens may also evolve adaptive strategies in response to elevated CO_2_. However, studies revealing elevated CO_2_ effects on insect herbivory and pathogen infections rarely used elevated CO_2_-adapted insects and pathogens. Thus, it is important to consider this issue to better understand the response of tea plants to pests and pathogens. Moreover, genotype screening in response to elevated CO_2_ is necessary to develop tea cultivars resilient to climate change.

Plant β-carbonic anhydrases (βCAs) are important CO_2_ sensing and metabolizing proteins that catalyze the interconversion between CO_2_ and ${\text{HCO}}_{3}^{-}$ (DiMario et al., 2017; Kazan, 2018). However, the role of βCAs in tea plants under elevated CO_2_ remains far from being substantiated. Multiple hormones and their interaction mediates plant response to biotic and abiotic factors. Although a few studies have highlighted a role for gibberellins, abscisic acid, brassinosteroids, and SA in tea quality, the role of hormones and signaling molecules in elevated CO_2_-induced changes in tea quality remains largely unknown. In addition, major transcription factors, such as, MYB, WRKY, bHLH, and WD40 that regulate the biosynthesis of terpenoids and flavonoids in other plants, should be taken into account to explore the mechanisms of elevated CO_2_-induced regulation of tea quality.

In summary, existing literature suggests that elevated CO_2_ promotes tea yield and quality, but it attenuates tea plant resistance to some insects and pathogens, which poses a serious threat to future tea production systems. Tea quality is a complex perception of multiple factors, which is believed to be improved under elevated CO_2_ as tea plants grown under elevated CO_2_ accumulate high levels of catechins and theanine, and low level of caffeine in tea leaves. It appears that decreased caffeine accumulation under elevated CO_2_ is one of the main reasons of attenuated defense response in tea plants. However, there are some technical limitations in the studies relating to elevated CO_2_ effects on tea plants, which should be taken into account to design more realistic experiments to better understand the responses of tea plants to elevated CO_2_ in the future.

## Author Contributions

GA and XL conceived and designed the manuscript, and wrote the draft manuscript. GA, XL, AL, and SC reviewed and edited the manuscript. All authors have read and approved the manuscript.

## Conflict of Interest

The authors declare that the research was conducted in the absence of any commercial or financial relationships that could be construed as a potential conflict of interest.

## Footnotes

1 http://www.esrl.noaa.gov/gmd/ccgg/trends/
